# Editorial: Functional and innovative food ingredients: assessment of analytical, microbiological and sensory aspects

**DOI:** 10.3389/fnut.2023.1207323

**Published:** 2023-05-10

**Authors:** Roberta Foligni, Andrea Pulvirenti, Luciana De Vero, Cinzia Mannozzi

**Affiliations:** ^1^Department of Agricultural, Food and Environmental Sciences (D3A), Università Politecnica delle Marche, Ancona, Italy; ^2^Unimore Microbial Culture Collection Laboratory, Department of Life Sciences, University of Modena and Reggio Emilia, Reggio Emilia, Italy

**Keywords:** bioactive compounds, metabolic syndrome, obesity, chronic inflammation, oxidative stress

The food industry is characterized by a high level of innovation, primarily due to consumers' concern about their choice on food and their possible health and environmental implications.

A lot of research has been performed in this field, focusing on the increase of food quality in all its facets. Suitable starter cultures are fundamental for developing novel fermented products due to their health-promoting functions (1). For instance, some bacteria and yeasts species are able to detoxify mycotoxins (2). Concerning to sensorial aspects, some yeasts have a marked enzymatic activity and can be exploited for their potential ability to enhance the aroma profiles (3).

Another important aspect is the chemical composition, which has been analyzed in different projects with the aim of characterizing a local variety of food ingredients. For example, some researchers (4) made a comparison between fatty acid profiles of old and modern varieties of *Triticum turgidum* and *T*. *aestivum* collected in the Marche region, assuming the existence of a genetic base for the differences in whole fatty acid profile between old and modern wheat. As well as Boselli et al. (5) analyzed the correlation between the antioxidant power of the ethanolic truffle extracts and their chemical composition determined by high-resolution mass spectrometry. An innovative field of research is the study of a food ingredient with a bad reputation among consumers, to find whether or not their opinion is based on scientific data. A perfect example can be found in a review about hybrid palm oils (6), where the authors claimed that the overconsumption of this hybrid should be carefully monitored as the original one. The innovation can also regard the use of an ingredient already known, but used in a new production, for example, it has been exploited the sea fennel (*Crithmum maritimum L*.) to realize high value preserves at the industrial level with selected starters (7).

This editorial aims to summarize the Research Topic studies involving innovative food ingredients, their production process, and their possible application in the food industry. These ingredients have different beneficial effects on food (improving quality and nutritional features) and on the human body (antibacterial activity, antitumoral…).

Andersen et al. analyzed the improvement in the nutritional value of different Caciotta-like cheeses by adding in their based formulation blackcurrant (*Ribes nigrum*) and Cornelian cherry (*Cornus mas*). These ingredients have been tested according to three variables: the production conditions (conventional vs. organic), the processing conditions (freeze-dried and not), and the amounts used (0.3 or 0.6%). The authors concluded that both the additions of Cornelian cherry and blackcurrant determine an increase of the total polyphenol content without affecting the cheeses, except for the appearance feature. Furthermore, a positive correlation between the addition of blackcurrant and the growth of lactic acid bacteria was observed.

Buniowska-Olejnik et al. explored the use of turmeric extract in the formulation of probiotic yogurt. The influence of this additive on the growth of probiotic bacteria, its antibacterial activity, and its use as a quality indicator of low-fat yogurt have been investigated. Their work demonstrated that the water-dispersible form of this ingredient showed antimicrobial activity against pathogenic microorganisms, especially Gram+ bacteria and fungi. It also supports the development of the probiotic culture, and improves the product's color, increasing its attractiveness. Finally, the turmeric extract ensures the stability of the product's quality and safety during 28 days of storage (Buniowska-Olejnik et al.).

Skalickova et al. made a comparison of the nutrient content of pea flour after cooking and lactic fermentation with *Lactobacillus plantarum*, before and after digestion *in vitro* at different combinations of time and temperature. The nutrient analysis showed that lactic fermentation increased the amino acid content, reduced the presence of sugars, and improved the bioaccessibility of micronutrients like Mn and Fe. On the other hand, the cooking process caused a general decrease in the polyphenols content and mineral concentration. None of the applied processes affected phytic acid concentration and phytase activity (Skalickova et al.).

Wang et al. produced a review about all the biological activities of chlorogenic acid (CGA) and determined its application in the food and food packaging industry. This ingredient was found to have a comprehensive antioxidant activity thanks to the hydroxyl and carboxyl groups of its structure. It has also a good protective effect on the liver and kidney and can prevent tumor growth and liver diseases by regulating the expression of enzymes and proteins related to the oxidation system and controlling cell apoptosis. Chlorogenic acid has also an antimicrobial activity both on Gram-positive and negative bacteria. It can inhibit the synthesis of bacterial cell membranes, resulting in the loss of cell contents and bacterial inactivation. Moreover, it plays a role in regulating glucose and lipid metabolism, alleviating the development of diabetes, and reducing fatty acid deposition. It can also modulate the expression of pro-inflammatory and anti-inflammatory factors and hinder various damage caused by inflammation to the body. Finally, chlorogenic acid can protect the nervous system, prevent the progression of Alzheimer's disease, and has a potential antihypertensive effect. Thanks to all these biological properties chlorogenic acid can be used in many ways in the food industries. It can be used as an emulsifier with protein hydrolysates, a dye combined with acid-flavonol-anthocyanin and thanks to its anti-bacterial effect could be a valuable food preservative. Furthermore, it can help to extend the shelf-life by limiting lipid oxidation and change the protein structure decreasing the allergenicity of specific food compounds. Chlorogenic acid might have great potential on packaging materials in combination with others compounds such as chitosan or starch. Lastly, some researchers have used this compound as a natural antioxidant incorporated into the formulation of functional foods, and as a prebiotic to stimulate the growth of beneficial bacteria (Wang et al.).

All the articles of this Research Topic open new frontiers on food ingredients and their possible application, with the common goal of increasing food quality and safeguarding consumer welfare.

## Author contributions

RF: conceptualization, writing—original draft, writing—review and editing, and supervision. AP, LD, and CM: writing—review and editing and supervision. All authors contributed to the article and approved the submitted version.
